# Male descendant kin promote conservative views on gender issues and conformity to traditional norms

**DOI:** 10.1017/ehs.2021.29

**Published:** 2021-05-28

**Authors:** Nicholas Kerry, Khandis R. Blake, Damian R. Murray, Robert C. Brooks

**Affiliations:** 1 Department of Psychology, Tulane University, 2007 Percival Stern Hall, New Orleans, LA 70118, USA; 2 School of Psychological Sciences, The University of Melbourne, Australia; 3 Evolution and Ecology Research Centre, The University of New South Wales, Sydney, Australia

**Keywords:** gendered fitness interests, inclusive fitness, motivated cognition, gender roles, political attitudes, conservatism

## Abstract

Political and social attitudes have been shown to differ by sex in a way that tracks individual self-interest. We propose that these attitudes also change strategically to serve the best interests of either male or female kin. To test this hypothesis, we developed a measure of gendered fitness interests (GFI) – an index which reflects the sex, relatedness and residual reproductive value of close kin. We predicted that people with male-biased GFI (i.e. people with more male kin of a reproductive age) would have more conservative attitudes towards gender-related issues (e.g. gender roles, women's rights, abortion rights). An online study using an American sample (*N* = 560) found support for this hypothesis. Further analyses revealed that this relationship was driven not only by people's own sex and reproductive value but also by those of their descendant kin. Exploratory analyses also found a positive association between male-biased GFI and a measure of conformity, as well as a smaller association between male-biased GFI and having voted Republican in the last election. Both of these associations were statistically mediated by gender-related conservatism. These findings are consistent with the hypothesis that GFI influences sociopolitical attitudes.

## Graphical abstract

**Figure:**
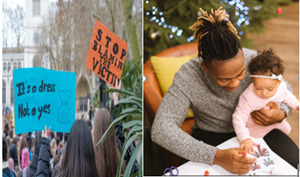

**Social media summary:** People with more male descendants of a reproductive age had more conservative attitudes on gender-related issues.

Attitudes towards sex, reproduction and gender roles differ between women and men (Ekehammar & Sidanius, 1982; DeVaus & McAllister, 1989; Luberti et al. 2020), and between parents and non-parents (Kerry & Murray, 2018, 2020). These attitudes also change with age (Cornelis et al., 2009) and with socioeconomic status (Petersen et al., 2013; DeScioli et al., 2014). Demographic differences in political attitudes have often been explained in terms of ‘motivated cognition’, such that people are motivated to develop political attitudes that optimise their self-interest (e.g. Kruglanski, 1996; Weeden & Kurzban, 2016; Jost, 2017). However, is it possible that such motivated cognition is also biased towards optimising one's inclusive fitness? Here, we investigate how one's proportion of male vs. female kin influences gender-relevant sociopolitical attitudes. Further, we hypothesise that the residual reproductive value of these kin (operationalised as age), as well as their relatedness to the focal individual, will also shape this relationship.

Attitudes that are considered ‘conservative’ often tend to support the social status quo and uphold moral tradition. In the USA (and many other countries), men have historically held more political power than women. For example, women's suffrage only came into law as recently as 1920, and the USA is still yet to elect a female president. Accordingly, many gender-related norms that are considered conservative tend to favour men or limit the freedoms of women. For example, modern conservatives are much less likely to favour women's choice relating to abortion, tend to support social structures in which men are afforded more power than women and are less likely to support equal opportunities for women in the workplace (e.g. Freeman, 1993; Pew Forum, 2019; Wolbrecht, 2002). We hypothesise here that people may have evolved to track social systems that benefit their inclusive fitness (i.e. the ability of an individual to pass genes on to the future generations, *including* those passed on by close relatives). Further, we propose that people with more of their inclusive fitness invested in the outcomes of females – i.e. not only women themselves, but people who have more female members of family with reproductive potential – will place a greater weighting on the interests of women, and will therefore be less likely to endorse conservative views on gender-related issues.

Alexander (1982, 1987) first observed that differences in reproductive interests between individuals generate some of the most important ethical, moral and ideological tensions that societies face. Betzig and Lombardo (1992) extended this line of thinking beyond individual reproductive interests to the fitness of relatives. They showed that people with more female kin in the 15–50 age group – in other words, those who were ‘at risk’ of unwanted pregnancy and the potential need for abortion – were more likely to hold ‘pro-choice’ views. Conversely, those with more reproductive age male kin were more likely to hold ‘pro-life’ policies.

A body of evidence suggests that these effects reflect the fact that the sex of a person's children covaries with their sociopolitical attitudes (reviewed by Lundberg, 2005). Policies enacted by firms whose CEOs have daughters tend to be more progressive and female-friendly (Cronqvist & Yu, 2017; Dasgupta et al., 2018). Parents of daughters express greater support for feminism and gender equity policies than those who have only sons (Warner, 1991; Warner & Steel, 1999; Prokos et al., 2010). Likewise, parents of daughters are more likely to vote for left-wing parties (Oswald & Powdthavee, 2010), or as elected representatives, to support bills concerning reproductive rights, working family support and tax-free education (Washington, 2008).

However, previous work has been inconsistent in operationalising offspring gender – with some studies counting all children and reporting ratios of sons and daughters (e.g. Oswald & Powdthavee, 2010) and others comparing people with sons or daughters with people with none (e.g. Warner, 1991; Dasgupta et al., 2018) and, in some cases, using combinations of these measures (e.g. Warner, 1991; Oswald & Powdthavee, 2010). Further, all of these studies have used archival data, and some of them have only found effects in subsamples (e.g. Prokos et al., 2010 – only in employed women; Shafer & Malhotra, 2011 – only in men). Meanwhile, other researchers have failed to find similar effects (despite large sample sizes), with one study finding effects in the opposite direction to those reported here and another finding null effects despite using two large samples (Conley & Rauscher, 2013; Lee & Conley 2016). Thus, while previous work broadly supports the idea that having more daughters vs sons leads to more liberal attitudes, questions remain regarding the robustness and consistency of effects, and regarding the best (i.e. most predictive) way to examine the effects of offspring gender.

Recent theoretical and empirical work has proposed that an individual's sociopolitical attitudes will track ‘gendered fitness interests’ (GFI) – the extent to which the individual's future genetic fitness is more likely to be a product of the reproductive success of living female vs male relatives (Blake et al., 2018; Brooks & Blake, 2021). This approach is grounded in both inclusive fitness (Hamilton, 1963, 1964a, b) and life history perspectives (Roff, 1992; Stearns, 1992). The GFI weights the contribution of a relative to the focal individual's GFI by their genetic relatedness (i.e. probability of sharing a given allele by descent) and their residual reproductive value (i.e. their age-based potential for future offspring; Brooks & Blake, 2021).

Some initial work has found evidence that GFI predicts social attitudes. Blake et al. (2018) tested the prediction that GFI could influence attitudes to mandatory Islamic veiling practices in Tunisia. Consistent with theoretical predictions, men were more in favour of Islamic veiling practices than women, but mothers of sons favoured veiling significantly more than mothers of daughters (Blake et al., 2018). However, this line of research is in its infancy and, to date, the possible implications of GFI for political attitudes remain largely unexplored. In the present study, our central hypothesis was that a male-biased GFI would predict more conservative views on gender-related issues. Note: this prediction is specific to issues which would differentially affect men vs women and does not include all elements of conservatism. For example, we would not predict that people with more male-biased GFI should support free-market economics (except to the degree that such attitudes are influenced by political identity, which might in turn be influenced by GFI).

There are also theoretical reasons to suggest that conformity – especially pertaining to traditional values and norms – might favour men and people with more male-biased GFI. Conformity – in the sense of adhering to traditional norms and values – entails supporting existing societal structures. Given that men have traditionally held greater power in the USA, conformity might benefit them more (or to put it conversely, conformity may be detrimental to women, and individuals with female-biased GFI). This argument is consistent with the observations that men consistently score higher in social dominance orientation than women (Sidanius et al., 1994), and that conservatives tend to conform more readily than liberals (Sistrunk & Halcomb, 1969; Feldman, 2003; Jost, 2017; Mallinson & Hatemi, 2018). There is also some tentative evidence that families with sons are more conformist than those with daughters (Koerner & Cvancara, 2002; Koerner & Fitzpatrick, 2002), and that adult women with a brother hold more conformist norms than women with a sister (Brenøe, 2019). Thus, we also tested the hypothesis that male-biased GFI would be associated with greater conformity. It is important to note that ‘conformity’ is operationalised in this study as greater trust in established norms. Importantly, our measure of conformity does *not* refer to the tendency to do the same as others or to copy more frequently occurring behaviours, as is often operationalised elsewhere (e.g. Denton et al., 2020; Henrich & Boyd, 1998).

## Method

Six hundred participants were prepaid on the MTurk crowdsourcing website. Participants who had completed related studies from our laboratory were excluded from participation. We did not filter, or purposely oversample, participants by demographic group other than that participants had to be based in the USA and be at least 18 years of age. We received 613 completed surveys, of which 17 failed a simple attention check. Of the 596 participants who passed the attention check, 25 were discarded owing to obvious errors in reporting of relatives (e.g. reporting the maximum number in each age category) and a further 11 owing to missing data in one or more fields, leaving 560 participants, giving at least 80% power to detect a small correlation of *r* = 0.12 (40). Of these participants, 320 (57.1%) were female, and 241 (43.0%) were parents. The sample was somewhat more liberal than average for the USA: of the 418 participants who reported having voted for one of the two main political parties, 64.1% reported voting Democrat, vs 35.9% who voted Republican. Consistent with previous work (Kerry & Murray 2018, 2020), there was proportionately a greater number of parents among Republicans than among Democrats (57.3 vs 38.8%). Participants were aged 18–72 years (mean = 35.61, SD = 12.13). Data, syntax and materials for the study are available at: https://osf.io/j4kra/?view_only=ab876ea5a8194698be85aa123b0a92a6.

### Procedure and measures

#### Gendered fitness interest

The GFI is a function of the sex, reproductive value and relatedness of an individual and their close kin. We calculated separate values representing GFI through the self only, through descendants only, through both self and descendants and through non-descendants. Given that a person's relatedness to themselves is 1, the GFI for the self is simply a product of one's own sex and reproductive value (and is often closely related to sex). The GFI from other relatives is weighted according to relatedness (e.g. 0.5 for children and siblings, 0.25 for grandchildren, etc.). Thus, GFI from descendants is the summed product of the sex, residual reproductive value and relatedness of each of an individual's children and grandchildren. The GFI for non-descendants involves a similar calculation from siblings, nieces and cousins. To calculate these GFI values, we simply use the sex, age and relatedness of each participant and their family members.

Participants reported their own age, as well as the number of sons, daughters, grandsons, granddaughters, brothers and sisters in each of the following age categories: 0–14, 15–24, 25–34, 35–44, 45–54, 55–64 and 65+ years old. We calculated *n_i_*, the proportion of total reproductive value remaining (i.e. residual reproductive value/total reproductive value), based on the assumption that *n_i_* = 1 until 20 years old, and then declines monotonically over 30 years until it reaches 0 at 50 years old. [We assumed no sex difference in the relationship between *n_i_* and age for these calculations. In reality, rates of *n_i_* decline are likely to be different in men and women. However, in the absence of robust estimates of *n_i_* decline that justify a different assumption, we thought it best not to introduce a source of sex differences into the estimates of GFI that we present here. However, to check that we were not inadvertently biasing results in favour of our hypotheses, we also calculated versions of GFI based on more realistic fertility declines such that *n_i_* in women declined uniformly over 25 years (from 21 to 45) and in men over 40 years (from 21 to 60). These assumptions produced very similar GFI values for both self (*r* = 0.995) and descendants (*r* = 0.997). Rerunning the main analyses with these alternative measures of GFI made no substantive difference to results.] Participant *n*_self_ could be calculated to the year, but the *n_i_* of each relative was based on the mid-point of the respective bin for those bins within or overlapping with ages 21–49 years.

Participants also reported a count of their male and female cousins, and their nephews and nieces. Owing to the lack of age information, we assume *n*_cousins_ = *n*_self_, which is to say that an individual's cousins are, on average, the same age as they are. For nieces and nephews, we made the simplifying assumption that *n_i_* = 1. These assumptions add some obvious error, but that error is random with respect to our hypotheses.

We then calculated GFI for each class of *y* relatives (where *y* denotes the number of members in a particular group of relatives, e.g. ‘grandchildren’), including the participant (i.e. self) according to the following formula:
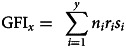
where *n*_i_ is residual reproductive value as a proportion of total reproductive value, as calculated above, *r*_i_ is the relatedness of descendant *i* to the participant, and *s_i_* is either −1 for females or +1 for males. We then summed the GFI estimates for children and grandchildren to create GFI_descendant_, and summed the GFI estimates for siblings, nephews/nieces and cousins to create GFI_non-descendant_. These two components of GFI, together with GFI_self,_ are used in the analyses below, and are also summed to create an overall estimate of GFI.

#### Gender-related conservatism

This measure was based on eight statements, adapted from The Longitudinal Study of Generations (Silverstein & Bengtson, 2008), which were designed to measure gender-specific aspects of political attitudes, such as gender roles and women's rights. Participants rated agreement on a seven-point scale (1 = strongly disagree, 7 = strongly agree) with statements such as ‘A woman should have the right to an abortion if she wishes’ and ‘If a woman and man both work full-time, they should share household tasks equally’ (both reverse scored). We extracted a single principal component from responses to the eight statements. The extracted principal component had an eigenvalue of 4.42 and comprised 55.2% of the original variance (see Supplementary Material Table S1 for full items and loadings). Higher positive values represent more conservative/traditional attitudes.

#### Conformity

Conformity was measured using a six-item scale developed by Murray and Schaller (2012). This scale measures conformity with traditional values and norms. It does not measure conformity in the sense of an inclination to adopt common behaviours. Participants read statements such as ‘Too many new ideas in one country can cause its values to erode’ and ‘Constantly breaking social norms often has harmful, unintended consequences’. Participants then rated their agreement with these statements on a six-point scale (1 = strongly disagree, 6 = strongly agree). As with the conservatism measure, a principal components analysis suggested a single-factor solution. This extracted component had an eigenvalue of 3.27 and accounted for 54.52% of variance. All items loaded positively with *β* ≥ 0.60. Note: the current data were collected as part of a wider project and included a measure of conformity to test a separate hypothesis.

#### Voting habits

Participants were simply asked ‘Which political party did you last vote for?’ with three options: ‘Republican’, ‘Democrat’ and ‘other/did not vote’. For the purposes of the analyses reported here, we dichotomised this variable into Republican and Democrat voters, with the third category omitted. In US politics, these two parties receive over 95% of all votes in general elections. The Republican Party is considered politically conservative and the Democrats politically liberal.

## Results

Bivariate correlations are presented in Table 1. Analyses were conducted using SPSS software, unless otherwise noted. As shown in the table, GFI through self plus descendants was positively associated with both gender-related conservatism (*r* = 0.19, *p* < 0.001, 95% CI [0.11, 0.28]) and conformity, (*r* = 0.14, *p* = 0.001, 95% CI [0.05, 0.23]). Based on these correlations, the GFI accounted for around 4% of variance in gender-related conservatism and 2% of the variance in the conformity measure. Although these relationships are relatively small, GFI was more strongly associated with either of these outcomes than other demographic predictors such as age, sex or number of offspring.

**Table 1.** Bivariate correlations between key variables

Variable	1	2	3	4	5	6	7
1. GFI_self+descendant_	—						
2. GFI_self_	0.80***	—					
3. GFI_descendant_	0.64***	0.04	—				
4. GFI_non-desc_	0.09*	0.07	0.05	—			
5. Conservatism	0.19***	0.17***	0.10*	0.01	—		
6. Conformity	0.14**	0.07	0.14***	0.02	0.59***	—	
7. Age	−0.02	0.00	−0.03	0.04	0.07	0.05	—
8. Total offspring	−0.03	−0.03	−0.01	−0.01	0.14**	0.09*	0.19***

Note: *** *p* < .001, ** *p* < .01; * *p* < .05.

Further, these relationships are not simply explained by GFI through the self alone. Both gender-related conservatism and conformity correlated significantly with GFI from descendants only (*r* = 0.10, *p* = 0.014, 95% CI [0.02, 0.19]; *r* = 0.14, *p* = 0.001, 95% CI [0.05, .22], respectively). Non-descendant GFI was unrelated to either gender-related conservatism (*r* = 0.01, *p* = 0.75, 95% CI [−0.07, 0.10]) or conformity (*r* = −0.02, *p* = 0.65, 95% CI [−0.10, 0.06]). One reason for this may be that numbers of cousins and niece/nephews were more prone to measurement error than sons, daughters, grandchildren and siblings in our study owing to differences in the way we asked participants to provide input. Weak GFI effects are also consistent with models developed in previous research that found that non-descendant kin do not exert large effects on GFI under family sizes similar to those observed in our sample (Brooks & Blake 2021) because the many, small (owing to low relatedness [*r*], except in full siblings) contributions of non-descendant kin like cousins, aunts and uncles tend to sum towards zero. For brevity – and given that both mathematical models and the current data suggest little role for GFI from non-descendants – the results reported here focus on GFI from the self and from descendants.

We fitted a series of regression models to estimate the effects of GFI and its components, together with age and number of offspring, on our composite measure of gender-related conservatism (Table 2). We included age and number of offspring in the models, since both age and parenthood have been found to predict conservatism, especially on social issues (Cornelis et al., 2009; Kerry & Murray 2018, 2020). Throughout all of the models, conservatism increased with number of offspring and was not significantly affected by age. A regression model which included GFI through self plus descendants alongside the participant's age and number of children yielded a significant positive effect of GFI_self+descendant_ on gender-related conservatism, *b = 0.*25 (SE = 0.05), *p* < 0.00001, 95% CI [0.15, 0.35], with an independent effect of number of children, *b = 0.*14 (SE = 0.04), *p* = 0.001, 95% CI [0.06, 0.22], but no significant effect of age, *b = 0.*004 (SE = 0.003), *p* = 0.249, 95% CI [−0.00, 0.01].

**Table 2.** Regression models testing the effects of GFI and its components on gender-related conservatism, together with age and number of offspring.

Model	1	2	3	4	5	6
R^2^_Adjusted_	0.019	0.047	0.039	0.053	0.055	0.056
Age	0.004	0.004	0.004	0.004	0.004	0.004
	(0.004)	(0.003)	(0.003)	(0.003)	(0.003)	(0.003)
N Offspring	0.131**	0.137**	0.137**	0.139**	0.139**	0.138**
	(0.04)	(0.04)	(0.04)	(0.04)	(0.04)	(0.04)
					
GFI_self_		0.278***		0.272***	0.271***	
		(0.07)		(0.07)	(0.07)	
GFI_descendants_				0.210*	0.210*	
				(0.09)	(0.09)	
GFI_non-desc_				-0.005		
				(0.06)		
GFI_self+descendants_						0.248***
						(0.05)
GFI_total_			0.132***			
			(0.04)			

Note: Coefficients are unstandardized. *** *p* < .001, ** *p* < .01; * *p* < .05.

A linear regression analysis with GFI_self+desc_ as a predictor alongside age and number of children found an effect of GFI_self+desc_, such that a more male-biased score was associated with greater conformity, *b = 0.*18 (SE = 0.05), *p* < 0.001, 95% CI [0.07, 0.28], with no significant effects of number of children, *b = 0.*08 (SE = 0.04*)*, *p* = 0.051, 95% CI [−0.00, 0.17] and age, *b = 0.*003 (SE = 0.004), *β* = 0.04, *p* = 0.34, 95% CI [−0.00, 0.10].

Although the participants’ own sex is an important component of GFI, these relationships cannot be fully explained by it. The GFI – whether operationalised to include the self and descendants, or only descendants – was significantly positively associated with both gender-related conservatism and conformity, even when controlling for sex, age and total number of offspring (all values of *β >* 0.10, all values of *p* < 0.01).

Previous work has reported an association between offspring gender and voting habits (e.g. Washington, 2008; Oswald & Powdthavee, 2010; Conley and Rauscher, 2013). To test whether GFI was related to political party affiliation, we ran a bootstrapped binary logistic regression with GFI (through self plus descendants) as a predictor and the dichotomised political party affiliation as the dependent variable. Male-biased GFI was associated with greater likelihood of having voted Republican (i.e. conservative) in the last election, *b = 0.*34 (SE = 0.13), *p* = 0.011, 95% CI [0.07, 0.60]. While the magnitudes of the relationships for self and descendants were similar, only GFI_self,_ independently reached significance, *b = 0.*37 (SE = 0.18), *p* = 0.031, 95% CI [0.03, 0.73], while the association with GFI_descendant_ was positive but nonsignificant, *b = 0.*32 (SE = 0.23), *p* = 0.151, 95% CI [−0.08, 0.82].

These relationships between GFI and voting behaviour are relatively small, but may be of practical significance. To put these effects in more interpretable terms, we created a *Z*-score for GFI_self+desc_ and split participants into four groups based on these *Z*-scores. Of those with GFI scores at least 1 SD below average (i.e. the most female-biased GFI), 75% of those who reported voting (*n* = 72) said that they had voted Democrat. Of those people whose *Z*-scored GFI was between −1 and 0 and reported voting (*n* = 179), 68.6% voted Democrat. For those between 0 and +1.0 (*n* = 117), this further decreased to 57.3%, and for those (*n* = 70) who scored at least 1 SD above average, the proportion was 54.3%.

### Mediation analyses

We hypothesised (*post-hoc*) that GFI-related differences in political affiliation and conformity exist to the extent that these attitudes (conformity and support of a conservative/liberal political party) support gender-related goals. This hypothesis implies that differences in gender-relevant conservatism would mediate the positive relationship between GFI and conformity and between GFI and political party affiliation. To test this, we ran a mediational model using the LAVAAN package (Rosseel, 2012) in the R programming environment with GFI as the predictor, conformity as the outcome variable and conservatism as the mediator. This yielded a significant indirect effect of GFI through conservatism, *b* = 0.14 (SE = 0.03), 95% CI [0.08, 0.21], but no direct effect of GFI, *b* = 0.04 (SE = 0.04), 95% CI [−0.05, 0.12]. The total effect for the model was also significant, *b* = 0.18 (SE = 0.05), 95% CI [0.07, 0.28]. This indirect effect was also robust to the inclusion of age and total number of offspring as covariates.

To examine whether this mediation effect was simply a result of the covariances between variables, we ran an alternative model with conformity as the mediator and conservatism as the outcome. While this second model still yielded a significant indirect effect, this indirect effect was smaller than in the first model (42% vs 78% of total effect), *b* = 0.10 (SE = 0.03), 95% CI [0.04, 0.16], and there remained a stronger, and highly significant, direct effect of GFI on gender-related conservatism, *b* = 0.14 (SE = 0.04), 95% CI [0.05, 0.22]. The total effect for this model was also significant, *b* = 0.24 (SE = 0.05), 95% CI [0.14, 0.34].

A model in which GFI predicts party affiliation with gender-related conservatism as the mediator also found a significant indirect effect, *b* = 0.14 (SE = 0.04), 95% CI [0.07, 0.21], but no significant direct effect, *b* = 0.05 (SE = 0.08), 95% CI [−0.10, 0.21]. The total effect for the model was positive and significant, *b* = 0.19 (SE = 0.08), 95% CI [0.03, 0.36]. Switching gender-related conservatism and party affiliation revealed a highly significant direct effect, *b* = 0.16 (SE = 0.06), 95% CI [0.04, 0.28], with a smaller, but still significant, indirect effect, *b* = 0.10 (SE = 0.05), 95% CI [0.01, 0.19], and a significant total effect, *b* = 0.26 (SE = 0.06), 95% CI [0.13, 0.39]. Again, although this model does not demonstrate directionality, it shows that mediational effects are stronger (representing 74% vs 38% of total effects) when gender-related conservatism is the mediator, and is consistent with an account in which any effects of GFI on political party affiliation are contingent upon effects on gender-related conservatism. Further, these analyses demonstrate that GFI is associated with gender-related conservatism even when accounting for political party affiliation.

### Can these effects be better explained by alternative measures of offspring gender?

For comparison, we tested five alternative measures of offspring gender separately in a series of multiple linear regressions. These measures – total number of sons, the total number of daughters, the subtracted difference in number of sons and daughters and two dichotomous variables indicating whether or not a person had any sons and whether or not they had any daughters – were each included as predictor variables, alongside age and total number of children. Each of these analyses was run once with gender-related conservatism as the dependent variable, once with conformity and once (as a binary logistic regression) with political party affiliation. None of these measures of offspring gender were significantly associated with gender-related conservatism or political party affiliation after controlling for age and total number of children (all values of *p* > 0.17, without adjustment for multiple comparisons), and only number of sons was significantly associated with conformity, *b* = 0.20 (SE = 0.09), *p* = 0.035, 95% CI [0.01, 0.39]. However, given that 15 analyses were run, one instance of *p* < 0.05 is exactly what would be predicted by chance alone. Thus, the GFI from self and descendants (or either of these GFI components individually) was a more robust predictor of the outcome variables (gender-related conservatism and conformity) than previously employed measures of offspring gender.

These findings do not necessarily contradict previous work which found associations with these variables (e.g. Warner, 1991; Prokos et al., 2010; Oswald & Powdthavee 2010); some of these studies found small effects which would require greater statistical power to detect than the current sample allows. Further, it is worth noting that some previous work has operationalised ‘children’ differently to our definition here, using additional information which we did not collect (e.g. Lee & Conley, 2016 included only households in which all children were still living with their parents, and examined only the sex of the first child). Thus, we cannot make a fair comparison wit all existing literature. However, these analyses suggest that GFI may be a more robust and powerful predictor of sociopolitical attitudes than these other measures.

### Testing alternative causal explanations

We hypothesise here that the gender of family members affects sociopolitical attitudes. An alternative explanation for the relationship between GFI and conservative sociopolitical attitudes is that the political attitudes cause differences in the age distributions of sons and daughters. For example, it is possible that more conservative parents may have wanted to have at least one son, and might have (whether consciously or not) employed a ‘stopping-rule’ whereby they continued to have children until they had at least one son. One way to test if this is the case is to examine the sex of the youngest children in each family. Given that we only collected age-range data for each family member (i.e. 0–14 years old), we were unable to calculate this for all participants. However, we were able to infer the sex of the youngest child for 161 of the 241 parents in our sample. Based on this subsample, we found no support for the hypothesis that the GFI–conservatism relationship could be explained by a stopping rule. Examining by political party (for which 128 of this subsample provided an answer), there was no evidence that people of different political affiliations were employing (differing) stopping rules. Among Democrats, 61.4% (43 vs 27) of youngest children were male, whereas 60.3% (35 vs 23) of youngest children were male among Republicans. There were small, non-significant relationships between the participant's youngest child being male and both gendered conservatism (*r* = 0.07, *p* = 0.38) and conformity (*r* = 0.12, *p* = 0.12). However, in this same subsample of participants, regression coefficients for GFI_self+desc_ predicting the two key outcome variables were not reduced by adding the sex of the youngest child as a predictor, either compared with the simple effect of GFI in the subsample of 161 or compared with the effect of GFI in the overall sample. In fact, adding this covariate slightly increased the size of the GFI coefficient for gender-related conservatism (from *b* = 0.24 [in the subsample] to *b* = 0.35) and for conformity (from *b* = 0.18 to *b* = 0.19).

Examining GFI through descendants only, we found that the strength of the association with gender-related conservatism increased when controlling for the sex of the youngest child (from *b* = 0.14 to *b* = 0.18), although the association between GFI and conformity decreased somewhat within this subsample (from *b* = 0.15 to *b* = 0.09). This suggests that – in this subsample, at least – the associations between GFI and gender-related conservatism was not the result of a gender-based stopping rule. However, it is possible that such a stopping rule may have contributed to the association between GFI through descendants and conformity.

It is also possible that the associations between GFI and conservatism could be driven by conservatives having different types of families in other ways. For example, conservative families might be larger overall, and girls in conservative families might tend to have more younger (vs older) siblings. Thus, to control more comprehensively for family composition, we ran a multiple regression which included GFI as a predictor along with age, number of offspring, number of grand-offspring, ratio of sons minus daughters (coded from −1 to +1 where −1 indicates only daughters, + 1 indicates only sons, and e.g. two sons and one daughter is represented as +0.33), and ratio of grandsons to granddaughters (coded the same as the last measure). Even controlling for these measures of family composition, GFI was significantly positively associated with gender-related conservatism, *b* = 0.25 (SE = 0.06), *p* < 0.001, 95% CI [0.14, 0.36], as well as unique effects of number of offspring, *b* = 0.13 (SE = 0.04), *p* = 0.002, 95% CI [0.05, 0.21] and – interestingly – number of grand-offspring, *b* = 0.17 (SE = 0.07), *p* = 0.012, 95% CI [0.04, 0.30], but not effects of age, gender ratio of offspring, or gender ratio of grand-offspring (*p* > 0.75). Similarly, running a multiple regression with the same covariates, but with GFI from descendants only as the focal predictor, yielded a significant association with gender-related conservatism, *b* = 0.23 (SE = 0.10), *p* = 0.024, 95% CI [0.03, 0.43]. The same model also showed unique effects of number of offspring, *b* = 0.12 (SE = 0.04), *p* = 0.003, 95% CI [0.04, 0.21] and number of grand-offspring, *b* = 0.17 (SE = 0.07), *p* = 0.010, 95% CI [0.04, 0.30], but no effects of other predictors (*p* > 0.48).

The association between GFI and conformity was also robust to the same set of control variables with a significant association between conformity and GFI_self+desc_, *b* = 0.15 (SE = 0.06), *p* = 0.007, 95% CI [0.04, 0.26]. The same model contained a unique effect of number of grand-offspring on conformity, *b* = 0.15 (SE = 0.07), *p* = 0.031, 95% CI [0.01, 0.28], with no other predictors showing unique effects (*p* > 0.08). Another model with these control variables also showed a unique effect of GFI from descendants only, *b* = 0.27 (SE = 0.10), *p* = 0.010, 95% CI [0.06, 0.47] as well as an effect of number of grand-offspring, *b* = 0.15 (SE = 0.07), *p* = 0.025, 95% CI [0.02, 0.28]. Effects of all other predictors were non-significant (*p* > 0.09). The full models for these four regression analyses are reported in the Supplementary Materials (Tables S2–S5). As well as showing that GFI effects are robust to several measures of overall family size and composition, these four exploratory analyses suggest that having more grandchildren may also be associated with sociopolitical attitudes. However, this latter observation was not predicted *a priori* and should be treated as an exploratory finding requiring further testing.

## Discussion

As predicted, participants with male-biased GFI showed more conservative attitudes regarding gender-relevant issues. Moreover, this association was driven by the contributions of both the participant's self and their descendant kin, suggesting that attitudes towards gender-related political issues may be influenced both by one's own sex and the sex of descendant kin. This is consistent with the hypothesis that motivated political cognition may extend beyond the direct self-interest of the individual: people's political attitudes may also be motivated by the interests of their descendant kin. We also found evidence for a relationship between GFI and conformity, and a less consistent association with political party affiliation, both of which were statistically mediated by differences in gender-relevant conservatism.

These findings support the broader hypothesis that some individual differences in gender-related political attitudes arise from likely future fitness returns through male vs female descendant kin. Accordingly, these results support the predictive utility of considering not only an individual's own sex, but also their residual reproductive value and the GFI contributions from children and grandchildren in shaping the individual's sociopolitical attitudes (Brooks & Blake, 2021). This finding extends the well-established view that an individual's personal interests shape their adoption of attitudes and ideologies (Adorno, 1950; Jost, 2017), and suggests that a similar rationale may apply to multigenerational fitness interests. In extending theories of motivated political beliefs in this way, the current findings also illustrate how the gendered component of an individual's identity is more complex than male/female binaries, and to some extent, may share elements with a continuous trait. Note that the effects of GFI through descendants were of similar size to the effect of GFI through the self, a component that is often strongly associated with the sex of the self. Of course, other sex differences, and gender identity differences, associated with lived experience, are likely to make contributions to sociopolitical attitude formation that are independent of the reported GFI effects.

The absence of significant non-descendant kin GFI effects suggests that gendered fitness interests may not extend to non-descendant relatives like siblings, aunts/uncles, cousins, nephews and nieces. The only other published test for GFI effects (Blake et al., 2018) was restricted to one generation of descendant kin (i.e. offspring). Thus, to date, there remains no evidence in support of the broader claim of GFI theory, that inclusive fitness effects owing to genetic relatives broadly construed can influence attitudes. Our finding is also consistent with simulation models suggesting that non-descendant kin exert only small effects on GFI under small-medium family sizes like those typical of twenty-first century USA (Brooks & Blake, 2021). It remains possible that the quality of participants’ reporting on non-descendant kin was lower than for children and grandchildren, and thus contained considerably more error. Likewise, we also did not have specific age data for most of the non-descendant kin we tracked here, thus introducing more noise. Nonetheless, based on the data presented here, along with the outcomes of theoretically based models, the evidence suggests that non-descendant GFI is not a strong predictor of attitudes.

The present findings shed new light on previously reported (but sometimes apparently contradictory) relationships of offspring gender with political party affiliation and associated voting habits (Oswald & Powdthavee, 2010; Conley & Rauscher, 2013). We found that a more male-biased GFI (from self and descendants combined) was associated with a greater likelihood of having voted Republican in the last US election. However, the relationship between GFI only from descendant kin was not significantly related to party affiliation, nor was either the number of sons or the number of daughters, when controlling for age and total offspring. Thus, we did not find clear evidence that GFI predicted voting habits over and above participants’ sex, or that the gender of a person's descendants was associated with voting habits (consistent with null effects in Lee & Conley, 2016). Further, the relationship between GFI_self+desc_ and party affiliation was significantly mediated by gender-related conservatism (but controlling for party affiliation barely diminished the association between GFI and gender-related conservatism). This suggests the possibility that the relationship between offspring-gender and voting habits may exist to the extent that voting for a conservative party is perceived to benefit men, or perhaps more pertinently, to the extent that it is perceived to be detrimental to the interests of women. Further research might directly address this hypothesis by measuring beliefs about the gender-specific benefits of political parties.

Our exploratory analyses also found evidence of a positive association between male-biased GFI and conformity to traditional norms. Although this was not an *a priori* prediction (the conformity measure was included in the study as part of another hypothesis), the association is consistent with theory and with several previous findings. Conservatives place greater emphasis on conformity, and are indeed more likely to conform than liberals (Sistrunk & Halcomb, 1969; Feldman, 2003; Jost, 2017; Mallinson & Hatemi, 2018). Our analyses also indicate that differences in gender-related conservatism may mediate the relationship between male-biased GFI and conformity.

One explanation for our results could be that parents of sons might acquire their greater conformity and more conservative views, and parents of daughters their less conformist and more liberal views, through interaction with those offspring. It remains eminently possible, however, that a simple calculus of descendant kin GFI might impart both conservatism and conformity without a shared history of experience and conversation. Consistent with this interpretation, finding out the sex of a new child at birth (Oswald & Powdthavee, 2010; Shafer & Malhotra, 2011), or *in utero* (Pogrebna et al., 2018), can effect a change in attitudes or behaviour. Nonetheless, the data presented here cannot distinguish between these two explanations, and further research may seek to establish whether this relationship is related to social variables such as the amount and quality of contact with specific kin.

One possible limitation of the present study relates to the precision of the data used to calculate GFI scores. Ages of close relatives were only recorded approximately (as groups such as 0–14 and 15–24, rather than as exact ages), which is likely to have increased measurement error. A potential consequence of this imprecise data is a reduction in effect sizes, since increases in measurement error tend to dilute true effects. It is also possible that this contributed to the null effects relating to non-descendant kin (for which ages were not recorded directly, but instead were estimated from the participants’ own ages). Thus, more precise age data would provide a more rigorous test of possible effects of non-descendant kin. Further, precise age data would have allowed a stronger test of alternative hypotheses. We found no evidence for an alternative causal hypothesis according to which the association of GFI with sociopolitical beliefs was the result of conservative parents being inclined to continue having children until they had a son. However, this alternative hypothesis could only be tested with around two-thirds of the sample of parents – more precise data would have allowed a stronger test.

In addition to improving measurement, future research might aim to investigate what mechanism links family composition to political attitudes. It is unlikely that people have evolved to respond to ratios of different genders among their descendants by changing specific political attitudes, so future work should aim to test which psychological mechanisms mediate this relationship. For example, do people differentially experience psychological closeness in relation to specific genders according to the GFI from their family members, and does this closeness, in turn, lead to the adoption of specific attitudes? Or could empathy be involved: for example, might people with female-biased GFI experience greater empathy towards females?

In conclusion, the current work offers new insight into the relationship between gender, familial relationships and sociopolitical attitudes. Although existing work has linked the sex of offspring to political interests, it has been limited by methodological inconsistencies and mixed results (e.g. Oswald & Powdthavee, 2010; Conley & Rauscher, 2013; Lee & Conley, 2016). The data here suggest that GFI is a stronger and more robust predictor of conservative gender attitudes than previous measures, and that GFI may influence more general attitudes related to conformity. While further research is necessary to more properly understand the nature of causal relationships and the mechanisms involved, the results presented here suggest that just as one's own gender is a predictor of sociopolitical attitudes, so too is the gender of one's descendants.

## Supplementary material

For supplementary material accompanying this paper visit https://doi.org/10.1017/ehs.2021.29. click here to view supplementary material

## Research transparency and reproducibility

Data, code and materials are available at https://osf.io/j4kra/?view_only=ab876ea5a8194698be85aa123b0a92a6
