# Supplementary material for: Male descendant kin promote conservative views on gender issues and conformity to traditional norms
Source: Evol Hum Sci. 2021 May 28;3:e34. doi: 10.1017/ehs.2021.29 (PMC10427321; doi:10.1017/ehs.2021.29)
Supplement: Supplementary file 1 [file S2513843X21000293sup001.docx]

Table S1.

Component Score Coefficients for the single extracted PC

| Item |  | Component Score  Coefficient |
| --- | --- | --- |
| Some equality in marriage is a good thing, but by and large, the husband ought to have the main say in family matters |  | 0.189 |
| *It goes against nature to place women in positions of authority over men* |  | *0.184* |
| A woman should have the right to an abortion if she wishes |  | -0.147 |
| *The increase in the number of women who work has led to a decline in the quality of family life* |  | *0.169* |
| Women who want to remove the word "obey" from the marriage service don't understand what it means to be a good wife |  | 0.192 |
| *The “women's liberation” ideas make a lot of sense to me* |  | *-0.145* |
| A woman who places more importance on her career than on being a mother is denying her true nature |  | 0.184 |
| *If the husband and wife both work full time, they should share household tasks equally* |  | *-0.122* |

Table S2.

GFI for self and descendants predicting gender-related conservatism.

R^2^_Adjusted_ = 0.06

| Predictor | *b* | *SE* | *p* | Lower CI (95%) | Upper CI (95%) |
| --- | --- | --- | --- | --- | --- |
| GFI_self+desc_ | .25 | .05 | .000 | .14 | .36 |
| Age | .00 | .00 | .951 | -.01 | .01 |
| #offspring | .13 | .04 | .002 | .05 | .21 |
| #grand-offspring | .17 | .07 | .012 | .04 | .30 |
| OS ratio | -.04 | .12 | .752 | -.27 | .19 |
| Grand-OS ratio | .06 | .31 | .850 | -.54 | .66 |

Table S3.

GFI for descendants predicting gender-related conservatism.

R^2^_Adjusted_ = 0.04

| Predictor | *b* | *SE* | *p* | Lower CI (95%) | Upper CI (95%) |
| --- | --- | --- | --- | --- | --- |
| GFI_desc_ | .23 | .10 | .024 | .03 | .43 |
| Age | .00 | .00 | .950 | -.01 | .01 |
| #offspring | .12 | .04 | .003 | .04 | .21 |
| #grand-offspring | .17 | .07 | .010 | .04 | .30 |
| OS ratio | -.02 | .13 | .884 | -.28 | .24 |
| Grand-OS ratio | .03 | .31 | .924 | -.58 | .64 |

Table S4.

GFI for self and descendants predicting conformity.

R^2^_Adjusted_ = 0.03

| Predictor | *b* | *SE* | *p* | Lower CI (95%) | Upper CI (95%) |
| --- | --- | --- | --- | --- | --- |
| GFI_self+desc_ | .15 | .06 | .007 | .04 | .26 |
| Age | .00 | .00 | .968 | -.01 | .02 |
| #offspring | .07 | .04 | .081 | -.01 | .16 |
| #grand-offspring | .15 | .07 | .031 | .01 | .28 |
| OS ratio | .13 | .12 | .278 | -.11 | .27 |
| Grand-OS ratio | .22 | .31 | .484 | -.40 | .83 |

Table S5.

GFI for descendants predicting conformity.

R^2^_Adjusted_ = 0.03

| Predictor | *b* | *SE* | *p* | Lower CI (95%) | Upper CI (95%) |
| --- | --- | --- | --- | --- | --- |
| GFI_self+desc_ | .27 | .10 | .010 | .06 | .47 |
| Age | .00 | .00 | .950 | -.01 | .01 |
| #offspring | .07 | .04 | .094 | -.01 | .15 |
| #grand-offspring | .15 | .07 | .027 | .02 | .28 |
| OS ratio | .06 | .13 | .676 | -.21 | .32 |
| Grand-OS ratio | .16 | .32 | .611 | -.46 | .78 |
